# Giant Infrarenal Abdominal Aortic Aneurysm Treated With Elective Endovascular Repair Using an Anaconda Bifurcated Device: A Case Report

**DOI:** 10.7759/cureus.106822

**Published:** 2026-04-10

**Authors:** Oscar F Vargas, Juliana Salcedo-Mesa

**Affiliations:** 1 Interventional Radiology, Hospital Departamental de Villavicencio, Villavicencio, COL

**Keywords:** abdominal aortic aneurysm, case report, endoleak, endovascular aneurysm repair, giant aneurysm, stent graft

## Abstract

Giant abdominal aortic aneurysms (GAAAs) are extremely uncommon and carry a markedly increased risk of rupture. Their complex anatomy presents significant challenges for surgical management. Endovascular aneurysm repair (EVAR) has emerged as an increasingly utilized alternative in appropriately selected patients with favorable anatomy. We present the case of a 72-year-old male presenting with a non-ruptured, 12.8 cm infrarenal fusiform GAAA, successfully managed with elective EVAR using an Anaconda LoPro90 trimodular bifurcated endograft system (Terumo Aortic, Inchinnan, Scotland). We describe the clinical and radiologic features, technical approach, intraoperative challenges, and 10-month follow-up. EVAR using a bifurcated Anaconda endograft is a feasible and safe option for anatomically suitable GAAA patients, even in the presence of complex features such as angulated necks and iliac involvement. Further studies are warranted to establish long-term durability and optimal management protocols for GAAA.

## Introduction

Abdominal aortic aneurysms (AAAs) affect 4-8% of men over 60 years of age. This condition represents a significant cause of cardiovascular morbidity and mortality [1]. Current guidelines recommend elective repair at ≥5.5 cm in men and ≥5.0 cm in women. This is because larger aneurysms are more likely to rupture. Endovascular aneurysm repair (EVAR) is preferred over open surgery due to its minimally invasive nature, lower perioperative mortality, and better five-year survival compared to open surgery [1,2].

While most AAAs are diagnosed and treated at smaller diameters, aneurysms with larger diameters are rare (<0.03% prevalence) and are associated with rupture risks approaching 50% [2,3]. Therefore, giant AAAs (GAAAs), defined as >10 cm, are extremely rare, and their complex anatomy often poses technical challenges. For example, an angulated neck refers to the sharp bend at the top of the aneurysm, making it difficult for the endograft to fit securely. Short landing zones are the areas where the endograft must attach to a healthy aorta. Iliac involvement occurs when the aneurysm affects the iliac arteries, which may complicate treatment [4].

These anatomical complexities have traditionally favored open surgical repair for GAAA management, and open repair remains more commonly described for GAAA [4]. However, recent advances in endograft technology and delivery systems have expanded the applicability of EVAR to increasingly complex anatomies. Despite this progress, published experience with EVAR for GAAA remains limited. Consequently, there is a need for additional evidence demonstrating the feasibility and outcomes of endovascular treatment in this challenging subset of patients, as EVAR remains a promising alternative, especially when anatomical conditions are suitable, and device customization is available [1,2,5].

We report the case of a 72-year-old male with a 12.8 cm infrarenal GAAA successfully managed with elective EVAR using an Anaconda bifurcated endograft system. This report aims to describe the clinical presentation, anatomical considerations, technical approach, and 10-month follow-up. Such contributions add to the growing body of evidence supporting EVAR as a viable alternative in anatomically suitable GAAA patients.

## Case presentation

A 72-year-old male with hypertension, prior stroke with left hemiparesis, and benign prostatic hyperplasia was brought to the emergency department after a syncopal episode. He had a known AAA diagnosis 20 years earlier, without prior intervention. On admission, he was hemodynamically unstable, with hypotension (88/45 mmHg), tachycardia (101 bpm), afebrile (37 °C), and alert (Glasgow Coma Scale 15). Physical examination revealed pallor, delayed capillary refill, a pulsatile abdominal mass measuring approximately 4 × 5 cm, and bilateral scrotal swelling with erythema and tenderness. Initial laboratory evaluation findings were respiratory alkalosis, normal oxygenation, and hyperlactatemia.

**Figure 1:**
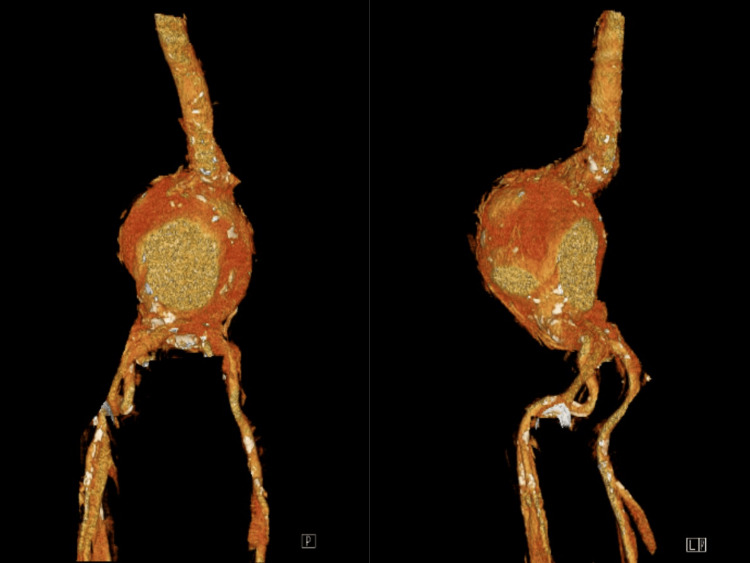
Contrast-enhanced 3D CTA of a giant infrarenal AAA 3D CTA showing a giant infrarenal abdominal aortic aneurysm (134 × 121 × 124 mm) with eccentric mural thrombus and a reduced central lumen, extending to the aortic bifurcation with involvement of both common iliac arteries. AAA, abdominal aortic aneurysm; CTA, CT angiography

**Figure 2:**
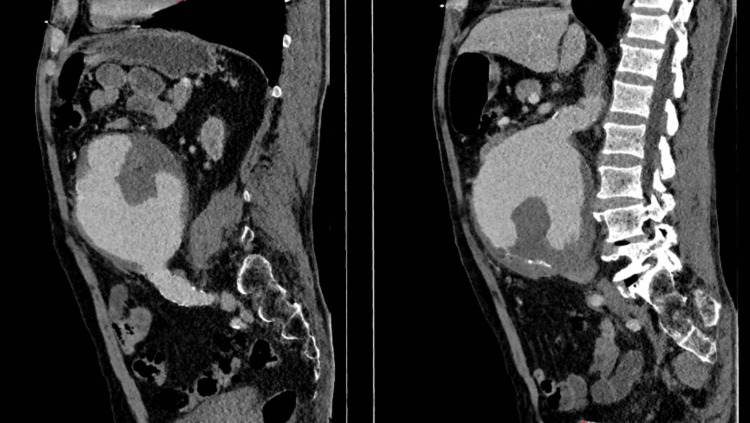
Contrast-enhanced CT (sagittal reconstruction) demonstrating a large infrarenal AAA Contrast-enhanced CT (sagittal reconstruction) showing a large infrarenal abdominal aortic aneurysm with eccentric mural thrombus and a centrally opacified residual lumen, without evidence of active contrast extravasation. AAA, abdominal aortic aneurysm

**Figure 3:**
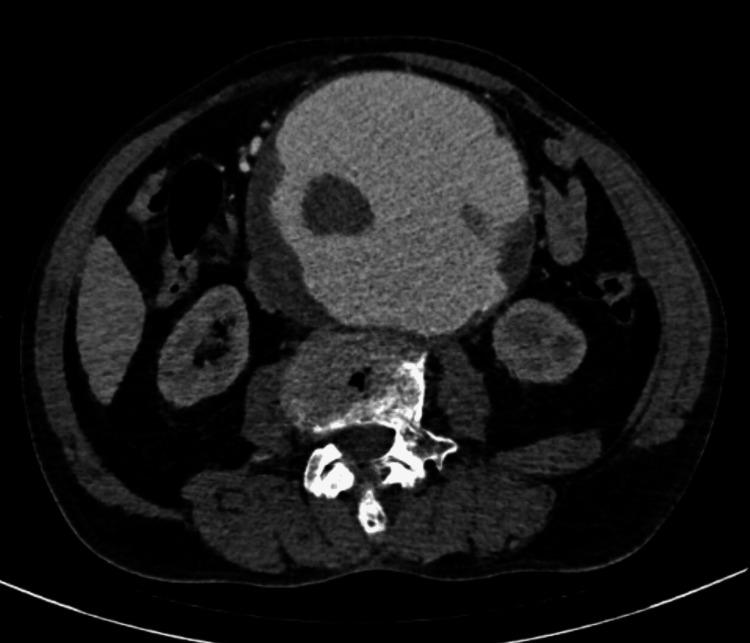
Contrast-enhanced axial CT of the abdomen Contrast-enhanced axial CT of the abdomen demonstrating a giant infrarenal AAA with extensive eccentric mural thrombus and a markedly reduced residual central lumen (true lumen measuring 86 mm in transverse diameter), features consistent with a high-risk aneurysm for imminent rupture. AAA, abdominal aortic aneurysm

An urgent CT angiography (CTA) revealed a giant infrarenal fusiform aneurysm measuring 134 mm (craniocaudal), 121 mm (anteroposterior), and 124 mm (transverse), with extensive mural thrombus and calcifications (Figure 1, Figure 2). The true lumen measured 86 mm in transverse diameter (Figure 3). There was no evidence of rupture. Additionally, aneurysmal dilation of the left common iliac artery (43 × 26 mm) was noted. Doppler ultrasound also showed bilateral orchiepididymitis.

The patient was admitted to the ICU, and uroculture revealed *Pseudomonas aeruginosa*. Intravenous ciprofloxacin and doxycycline were initiated. Given the aneurysm size and high rupture risk, urgent EVAR was planned after multidisciplinary discussion. Medical optimization included antihypertensives (losartan), beta-blockers (metoprolol), high-intensity statin (atorvastatin 40 mg), and analgesia.

Anatomic assessment and device planning

**Figure 4:**
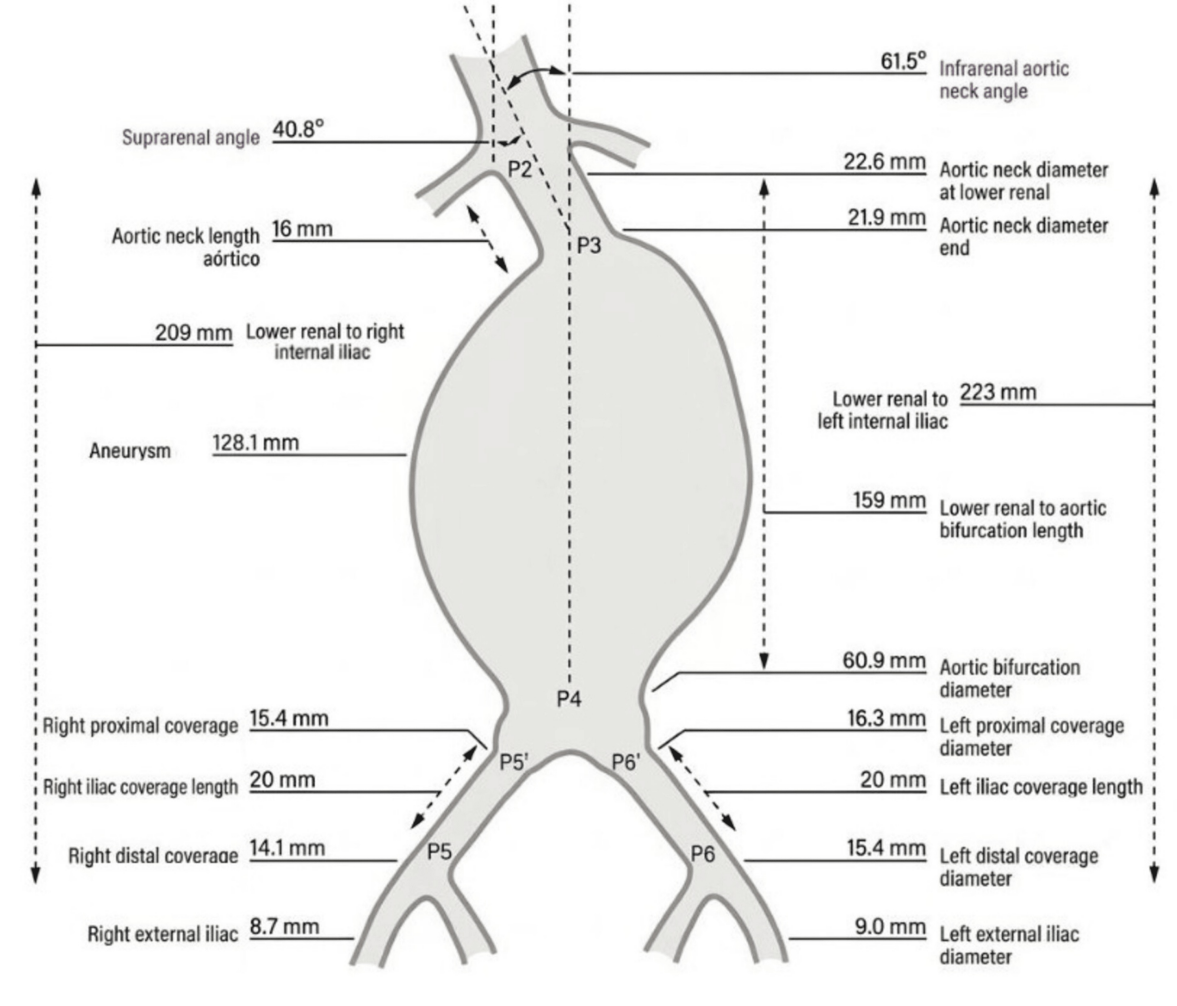
Anatomical measurements of GAAA for endovascular planning Anatomical measurements obtained from preoperative imaging used to guide device selection in EVAR. Key parameters are illustrated, including the proximal aortic neck diameter and length, which are critical for appropriate endograft sizing and fixation. EVAR, endovascular aneurysm repair; GAAA, giant abdominal aortic aneurysm

CT measurements showed an infrarenal angulation of 61.5°, conical neck morphology, and distances from the lowest renal artery to the left and right internal iliac arteries of 223 mm and 209 mm, respectively (Figure 4). An Anaconda LoPro90 trimodular bifurcated endograft system (Terumo Aortic, Inchinnan, Scotland) was selected: main body 25 mm, right limb 17 × 170 mm, left limb 19 × 130 mm.

Endovascular technique

Under general anesthesia and standard aseptic conditions, bilateral common femoral artery access was obtained using ultrasound-guided micropuncture, carefully avoiding anterior wall calcifications. Initial 7 Fr sheaths were upsized to 18 Fr (left) and 16 Fr Fortress introducer sheaths (Biotronik AG, Bülach, Switzerland) following pre-closure with two ProGlide devices per side (Abbott, Abbott Park, IL, USA).

**Figure 5:**
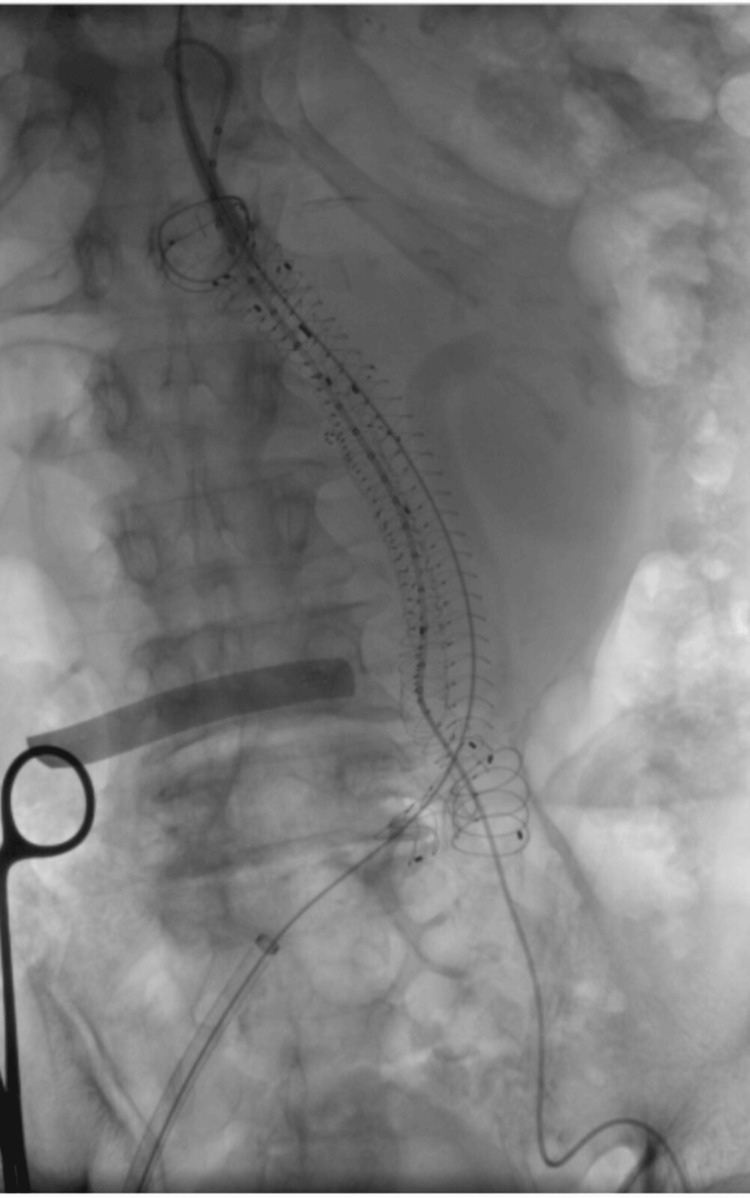
DSA during anaconda endograft deployment DSA was performed with a non-ionic contrast injection to identify the renal arteries, followed by the deployment of the Anaconda main body (28 × 40 mm) just below the lowest renal artery. DSA, digital subtraction angiography

A pigtail catheter was advanced from the left side to the level of the celiac trunk, while a multipurpose catheter was positioned from the right side into the aortic arch. Bilateral Supra Core stiff guidewires (Abbott) were placed. Digital subtraction angiography was performed using non-ionic contrast (15 mL/s at 600 PSI) to identify the renal arteries. The Anaconda endograft main body (28 × 40 mm) (Terumo Aortic) was deployed just below the lowest renal artery (Figure 5). Iliac extensions were deployed: left (19 × 150 mm and 10 × 130 mm) and right (17 × 170 mm).

**Figure 6:**
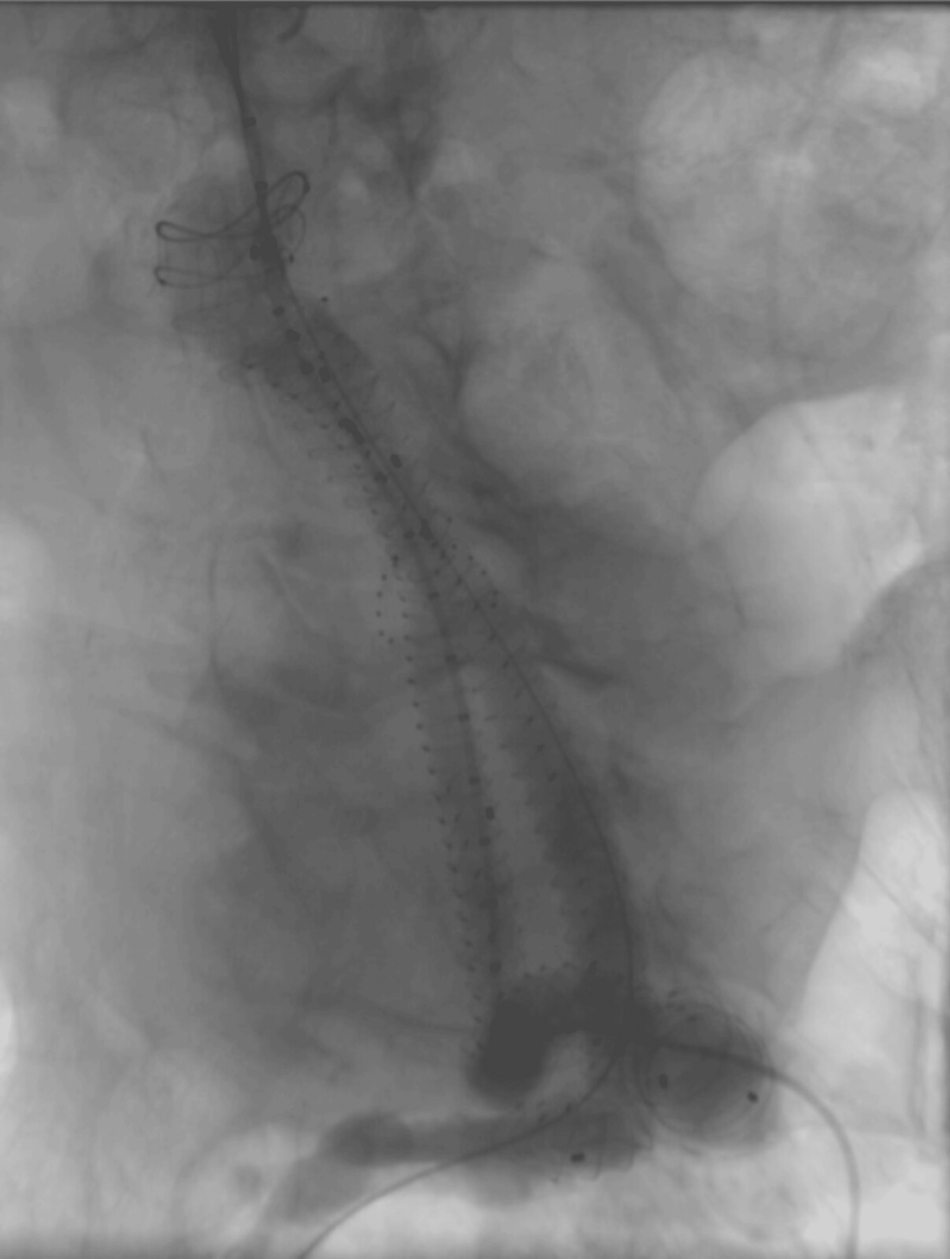
DSA during iliac extension deployment Iliac extensions were deployed to complete endograft positioning and ensure adequate distal sealing within the iliac arteries. DSA, digital subtraction angiography

A type IA endoleak was identified after initial deployment and was successfully managed with balloon angioplasty using a Reliant balloon catheter (Medtronic, Minneapolis, MN, USA) at the proximal sealing zone and iliac segments. Final angiography confirmed complete aneurysm exclusion, preserved renal perfusion, and no complications (Figure 6).

Femoral access sites were closed using the preplaced ProGlide devices. Total fluoroscopy time was 18.5 minutes, total radiation dose was 21,591 mGy, and 150 mL of contrast was administered.

Postoperative course

The patient tolerated the procedure well and was transferred to the ICU with antiplatelet therapy (aspirin 300 mg loading dose, 100 mg maintenance). He remained hemodynamically stable, with resolution of scrotal symptoms under antibiotic therapy. He was discharged on postoperative day 3 with statin, beta-blocker, angiotensin II receptor blocker, and aspirin.

**Figure 7:**
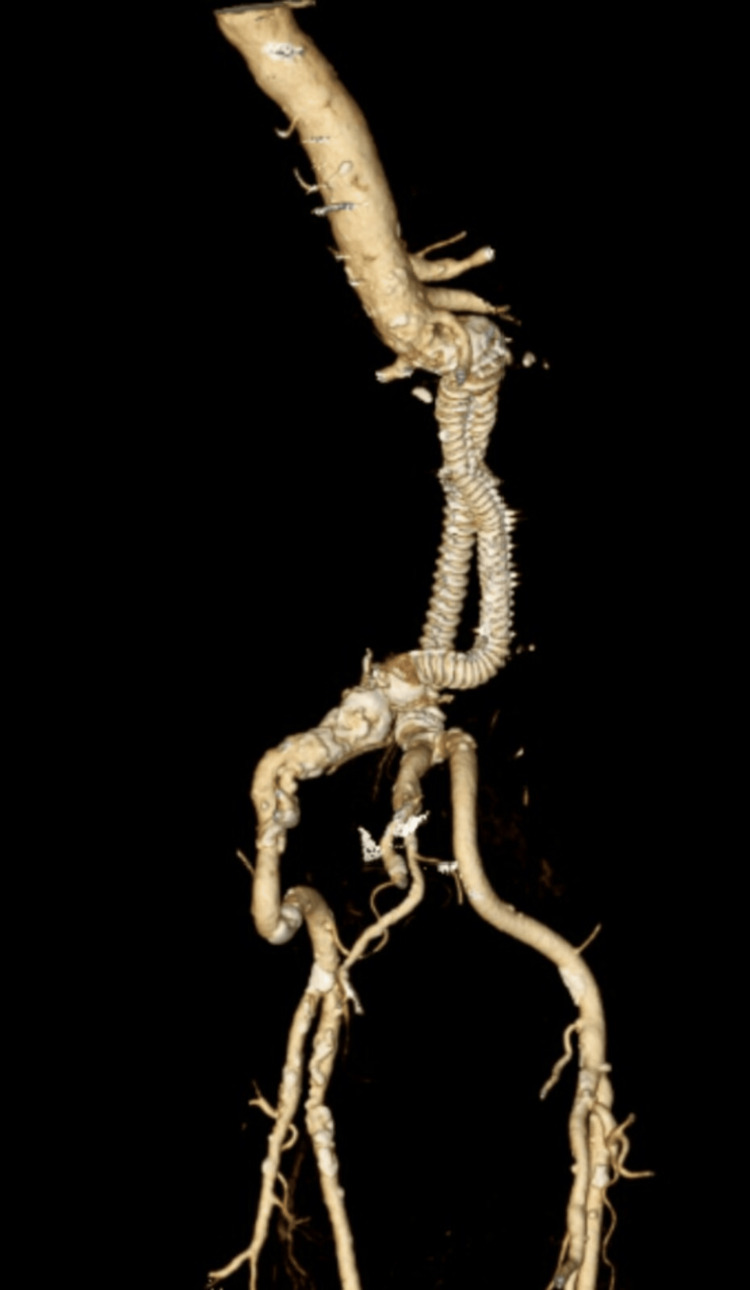
Follow-up CTA at 10 months post-EVAR Contrast-enhanced CTA demonstrating successful endovascular aneurysm exclusion. The Anaconda™ bifurcated endograft is well-positioned and fully patent, extending from the infrarenal aorta to the bilateral common iliac arteries. No endoleak is identified. Aneurysm sac measurements (139 × 124 × 116 mm) remain stable without significant interval change. No evidence of endograft migration, kinking, or limb compromise. CTA, CT angiography; EVAR, endovascular aneurysm repair

During the 10-month follow-up, the patient demonstrated a favorable clinical course, with no abdominal pain or complications. Follow-up CTA showed appropriate positioning of the endograft without evidence of endoleaks (Figure 7). The procedure was considered successful.

## Discussion

GAAAs represent an exceptionally rare but clinically significant subset of aortic pathology, with reported prevalence rates below 0.03% in population-based studies [2-4,6,7]. The natural history of GAAA is characterized by accelerated expansion rates and prohibitively high rupture risks, approaching 50% annually for aneurysms exceeding 8 cm in diameter [2,3]. Consequently, the optimal surgical approach remains a subject of ongoing debate, particularly given the complex anatomical features that frequently accompany these large aneurysms [8]. Our patient presented with several features traditionally considered unfavorable for endovascular repair: a maximum diameter of 12.8 cm, infrarenal neck angulation of 61.5°, conical neck morphology, extensive mural thrombus with calcification, and concomitant left common iliac artery aneurysmal disease. Historically, such anatomical configurations have favored open surgical repair [8]. However, the evolution of endograft technology has progressively expanded the envelope of anatomical suitability for endovascular approaches, incorporating design features specifically engineered to address hostile neck anatomy [9].

Although EVAR offers significant perioperative advantages (30-day mortality: 0.5-1.7% vs 3.0-4.7% for open surgery and a shorter hospital stay of two vs seven days), this early survival benefit diminishes after two to three years [10]. Reintervention rates are higher following EVAR (9.0% vs 1.7%), although most are minor endovascular procedures [10]. In this case, anatomical suitability and rapid planning enabled successful elective EVAR [2-4,6,7]. Key considerations included large aneurysm size, angulated neck, conical morphology, and iliac involvement. The Anaconda system’s flexibility and modular design facilitated adequate deployment in this complex anatomy [5,6,11]. Notably, the Anaconda LoPro90 system refers to a low-profile version with a 9 French delivery system, which facilitates access in patients with challenging iliac anatomy [12].

Lifelong surveillance is required in patients undergoing EVAR, as the risk of late rupture has been reported in up to 5.4% of patients, and the need for reintervention due to potential complications does not stabilize over time [12]. A hybrid follow-up strategy consisting of CT imaging during the first year followed by annual color duplex ultrasound is the most cost-effective approach in patients with less than 20% risk of contrast-induced nephropathy [13].

Regarding complications, iliac limb thrombosis represents one of the most significant complications associated with the Anaconda system [12]. Additionally, endoleaks may occur in 10-40% of patients undergoing EVAR [14]. These are classified into five types (I-V), with types I and III endoleaks requiring urgent management [14]. As observed in our case, type IA endoleaks, corresponding to inadequate proximal sealing at the aortic neck, can occur in approximately 6% of interventions and may be successfully managed with percutaneous transluminal angioplasty [8].

While this case report contributes valuable insights into the management of GAAAs, it is important to acknowledge several limitations. First, the single-case nature of this report limits the generalizability of the findings. Although the successful use of EVAR in this anatomically complex scenario is promising, it cannot be assumed that similar outcomes will necessarily be achieved in other patients with comparable anatomical challenges. Second, the relatively short follow-up duration raises concerns about the long-term viability of the treatment. Lifelong surveillance is recommended for EVAR patients due to risks of late rupture and other complications; therefore, outcomes reported in this case must be interpreted with caution until more extensive longitudinal data are available.

This report adds to the growing evidence supporting EVAR as a feasible and safe alternative, even in anatomically complex scenarios. Preoperative planning, endograft availability, and intraoperative imaging remain essential, and operator experience may be a more critical determinant of success than absolute anatomical parameters alone.

## Conclusions

This intervention highlights the successful performance of EVAR for a 12.8 cm infrarenal GAAA using an Anaconda bifurcated device, demonstrating feasibility in selected cases with complex anatomy, such as angulated necks and iliac involvement. This experience underscores the critical importance of preoperative planning, appropriate device selection, and operator expertise. These elements are essential in addressing the specific challenges presented by these cases. Despite the success of this procedure, remaining uncertainties include long-term durability of endografts in GAAA, optimal management of angulated necks, and endoleak risks. Further research is necessary to establish clear protocols for GAAA management and to evaluate the long-term outcomes of EVAR in these patients. Integrating these considerations will help advance clinical practice and improve care for these complex cases.
